# Enhanced Removal of Photoresist Films through Swelling and Dewetting Using Pluronic Surfactants

**DOI:** 10.1021/acs.langmuir.3c02034

**Published:** 2023-10-05

**Authors:** Masaki Hanzawa, Taku Ogura, Masaaki Akamatsu, Kenichi Sakai, Hideki Sakai

**Affiliations:** † NIKKOL GROUP Nikko Chemicals Co., Ltd., 3-24-3 Hasune, Itabashi, Tokyo 174-0046, Japan; ‡ Research Institute for Science and Technology, Tokyo University of Science, 2641 Yamazaki, Noda, Chiba 278-8510, Japan; § Department of Chemistry and Biotechnology, Faculty of Engineering, Tottori University, 4-101 Koyama-Minami, Tottori 680-8552, Japan; ∥ Department of Pure and Applied Chemistry, Faculty of Science and Technology, Tokyo University of Science, 2641 Yamazaki, Noda, Chiba 278-8510, Japan

## Abstract

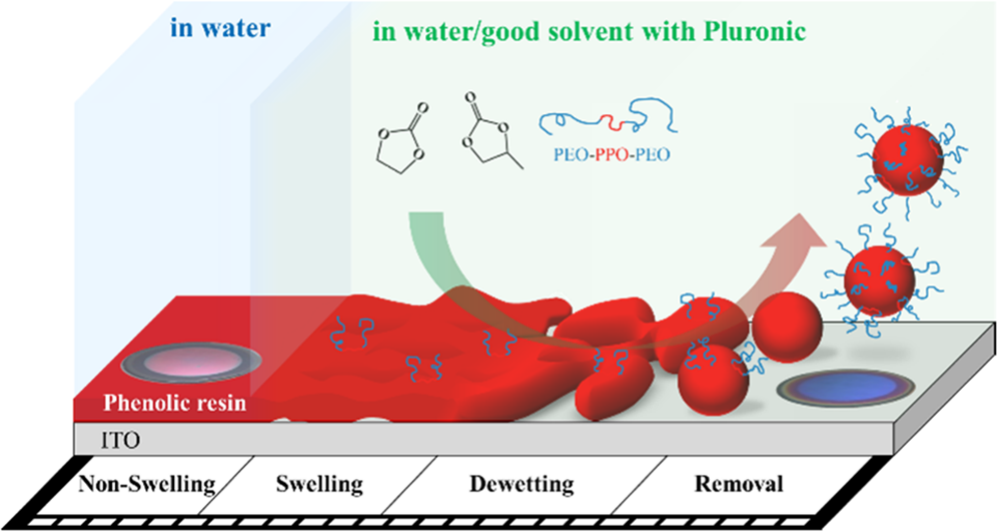

Organic photoresist coatings, primarily composed of resins, are commonly used in the electronics industry to protect inorganic underlayers. Conventional photoresist strippers, such as amine-type agents, have shown high removal performance but led to environmental impact and substrate corrosiveness. Therefore, this trade-off must be addressed. In this study, we characterized the removal mechanism of a photoresist film using a nonionic triblock Pluronic surfactant [poly(ethylene oxide)–poly(propylene oxide)–poly(ethylene oxide)] in a ternary mixture of ethylene carbonate (EC), propylene carbonate (PC), and water. In particular, the removal dynamics determined by using a quartz crystal microbalance with dissipation monitoring was compared with those determined by performing confocal laser scanning microscopy and visual observation to analyze the morphology, adsorption mass, and viscoelasticity of the photoresist film. In the absence of the Pluronic surfactant, the photoresist film in the ternary solvent exhibited a three-step process: (i) film swelling caused by the penetration of a good solvent (EC and PC), (ii) formation of photoresist particles through dewetting, and (iii) particle aggregation on the substrate. This result was correlated to the Hansen solubility parameters. The addition of the Pluronic surfactant not only prevented photoresist aggregation in the third step but also promoted desorption from the substrate. This effect was dependent on the concentration of the Pluronic surfactant, which influenced diffusion to the interface between the photoresist and the bulk solution. Finally, we proposed a novel photoresist stripping mechanism based on the synergy between dewetting driven by an EC/PC-to-water mixture and adsorption by the Pluronic surfactant.

## Introduction

Chemical cleaning technologies are widely utilized in various industries to achieve the desired functionalities and clean appearance.^1−3^ In addition to understanding the mechanical, chemical, and physical properties of contaminants in different scenarios, selecting an optimal cleaning method that avoids corrosion and discoloration of the substrate is crucial. Recently, the industry-wide demand for transitioning to chemical materials with low environmental impact has been increasing to achieve a sustainable society.^4^ However, this transition is challenging, particularly in the electronics industry, where removal performance and cycle time are prioritized in manufacturing processes. This is because even a residue of nanometer- to micrometer-sized structures can impair the original functions of electronic devices and reduce the yield.^5^ Therefore, the removal performance must not be compromised in the development of eco-friendly detergents.

Using polymeric coatings has been recognized as a conventional method of preventing unforeseen interactions, such as the degradation of art works,^6^ nonspecific adsorption of proteins,^7^ and metal corrosion,^8^ which modify the properties and structures of the surface. These coatings are designed to maintain their functionality for semipermanent use, whereas certain situations and purposes necessitate their removal.^6,9^ In particular, organic solvents and acid/base agents are effective in hydrophobic polymer removal; however, these substances often cause significant environmental impacts and can corrode metals. To address this issue, the application of nanostructured fluids is being investigated because such fluids offer effective cleaning with enhanced safety.^10,11^ This removal mechanism is facilitated by the combined action of suitable solvents and surfactants present in water.

Photoresist coatings, primarily composed of resins, play a crucial role in the manufacturing process of devices such as liquid crystal displays, smartphones, and tablet-personal computers.^12^ During lithographic light exposure, the photoresist coating forms a fine pattern on the substrate. Subsequently, the coating must be completely and quickly stripped from the substrate after etching protection. For high-throughput manufacturing, removal agents based on amine/organic solvent systems are commonly employed owing to their high removal rate and efficiency, despite their environmental impact.^13^ A combination of ethylene carbonate (EC) and propylene carbonate (PC) has emerged as a new photoresist stripper that leverages the chemical and biological properties of EC and PC.^13,14^ In our previous work, we investigated the antiadsorption mechanism of photoresist polymers on an indium tin oxide (ITO) substrate in an EC/PC mixture using nonionic triblock copolymers known as Pluronic surfactants [poly(ethylene oxide)–poly(propylene oxide)–poly(ethylene oxide); PEO_*x*_PPO_*y*_PEO_*x*_].^15^ Coating the ITO substrate with a brush layer of the Pluronic surfactant prevented interactions between the photoresist and the substrate. Small-angle X-ray scattering also revealed the formation of a core–corona structure between the photoresist and Pluronic surfactant (with the core representing the photoresist and the corona consisting of the Pluronic-PEO chain) in a mixture of EC/PC and water, as opposed to aggregation in the absence of Pluronics. This system in the presence of surfactants has the potential to serve as an effective and environmentally friendly cleaning agent for photoresist films.

A quartz crystal microbalance with dissipation monitoring (QCM-D) is a surface gravimetric analysis tool that offers high time resolution (within 1 s) and detection sensitivity (above 1 ng cm^–2^).^16^ Additionally, by simultaneously measuring energy dissipation, it provides valuable information about the viscoelasticity of the adsorbed layer. QCM-D can also be used to monitor the cleaning dynamics, as long as the shear wave is still present in the adsorbed film,^17^ and this technique has been applied in several recent studies.^11,18−20^ However, despite its compatibility with QCM-D, the removal dynamics in the electronics industry, which demands high-precision cleanliness, is still unclear. This emphasizes the potential of QCM-D as an analysis platform for evaluating new cleaning methods using interfacial technology. In addition, finding alternatives to stripping agents, which cause considerable damage to both substrates and the environment, is an unavoidable challenge for the electronics industry.

In this study, we characterized the removal mechanism of photoresist coatings from an ITO surface by using an EC/PC mixture and Pluronic surfactants. The photoresist cleaning data obtained by applying QCM-D were compared with those obtained by performing confocal laser scanning microscopy (CLSM) and visual observations. This comparison revealed the mechanism at different scales: the adsorption and viscoelasticity of the photoresist film at the nanoscale and the morphological changes at the macroscale. To gain further insights into the mechanism, we assessed the impact of the Pluronic surfactant concentration. Finally, we proposed a new photoresist stripper based on the synergistic effect of the EC/PC mixture and Pluronic surfactant. The novelty of this work is focusing on the mechanism by which polymeric films are removed in three components: good solvents, poor solvents, and Pluronic surfactants. This complex cleaning process is completed in a very short period of time, making it challenging to accurately capture the interfacial phenomena. Our findings have the potential to serve as an evaluation platform not only for electronics but also for wide varieties of industries with cleaning technologies such as textiles, metals, inks, toiletries, and households.

## Experimental Section

### Materials

Pluronic F-68, PEO_79_PPO_30_PEO_79_, was obtained from ADEKA (Tokyo, Japan). EC and PC were purchased from Kanto Chemical (Tokyo, Japan). Hexane, toluene, chloroform, ethyl acetate, methanol, ethanol, dimethyl sulfoxide, acetone, and fluorescent-probe Rhodamine B were purchased from FUJIFILM Wako pure chemical (Osaka, Japan). Fluorescent-probe Rhodamine 110 chloride was purchased from Sigma-Aldrich (Missouri). These materials were used without purification. The positive-tone photoresist (AZ SR-220) was obtained from AZ Electronic Materials (Luxembourg, Luxembourg), and it primarily consists of the novolak phenol-formaldehyde polymer and naphthoquinonediazide sulfonate as a secondary component. The photoresist material was dissolved in propylene glycol monomethylethyl ether acetate (PGMEA) at approximately 15% w/w. The F-68 concentration ranged from 0.1 to 1.0% w/w. EC and PC were mixed in a weight ratio of 70/30 (EC/PC).^21^ High-purity water was obtained using a Barnstead NANO pure Diamond UV system. The weight ratio of EC/PC (good solvent) to water (poor solvent) was set at 50/50 as the basic composition of the photoresist stripper (different ratio data are shown in the Supporting Information).

### Photoresist Film Formation

The photoresist film was prepared using the spin coating method as described in the literature.^15^ Prior to film formation, both an ITO-coated sensor (QSX 999, Biolin Scientific) and an ITO-coated soda-lime glass underwent sonication in ethanol and water for 10 min each. They were then dried under N_2_ gas and subjected to ultraviolet ozone treatment (SKB401Y-02, SUN ENERGY) for 10 min. The photoresist solution in PGMEA (volume: 20 μL) was added dropwise onto the cleaned ITO substrate. Subsequently, a “thin photoresist film” and a “thick photoresist film” were formed using a spin coater (ACT-220AII, ACTIVE Co., Ltd.): initially at a speed of 500 rpm (10 s) followed by 3000 rpm (10 s) or 1000 rpm (10 s) for the respective films. After solvent removal by heating at 130 °C for 5 min in a thermostatic bath (DVS403, Yamato Scientific Co., Ltd.), the photoresist films were ready for experimentation.

As used herein, the thickness of the film should be adjusted to accommodate the sensitivity of the instruments. For QCM-D measurements, it is preferable to have a thinner film to allow the shear wave to pass through without reflection at the film–bulk solution interface, which becomes more challenging as the film thickness increases.^17^ Conversely, a thicker film is advantageous for the irradiation of a visible light laser in CLSM. The spin coating conditions were determined based on the aforementioned considerations, resulting in the formation of a “thin photoresist film” (for QCM-D) with a thickness of several hundred nm and a “thick photoresist film” (for CLSM) with a thickness over several μm.

### Contact Angle Measurements

The static contact angle, θ, of a water droplet with a volume of 2.0 μL was measured by using a commercial contact angle meter (DropMaster 500, Kyowa Interface Co., Ltd.) equipped with a computer control system. This measurement aimed to assess the hydrophobicity of the photoresist film in comparison to the ITO substrate. The contact angle value was determined by using the θ/2 method. All experiments were performed at room temperature.

### QCM-D Measurements

The mass and viscoelasticity of the “thin photoresist film” in air and liquid were evaluated using a QCM-D instrument (QSense Explorer, Biolin Scientific). The liquid flow rate was set at 0.1 mL min^–1^; the selected overtones, *n*, were 1, 3, 5, and 7 for simultaneously measured resonant frequency, *F*_*n*_, and energy dissipation, *D*_*n*_, in this study. For low energy loss in the crystal oscillator from the overlayered film (that is, for the rigid and thin film), the adsorption amount, Δ*m*, is proportional to the shift, Δ*F*_*n*_, according to the Sauerbrey relation (eq 1).^22^
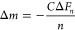
1Here, *C* is a constant of the sensitivity of the resonator (*C* = 0.177 mg Hz^–1^ m^–2^ for the 5 MHz quartz crystals). The shift, Δ*D*_*n*_, is the ratio between the dissipated energy (*E*_dissipated_) and the stored energy (*E*_stored_) in a single oscillation and is defined as follows (eq 2).
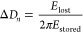
2

The penetration depth, δ, of acoustic shear wave in liquid or viscous media decreases as the resonant frequency increases.^23^ δ is calculated from the shear viscosity (η) and the density (ρ) of the overlayer and angular frequency (ω = 2*πF*_*n*_) according to eq 3, as follows.
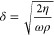
3As applied to QCM-D, multiple overtone analysis has the potential to provide insights into the spatial properties of the layer as well as the viscoelasticity of the material on top of the sensor surface; lower overtones provide information about the film further away from the sensor surface. The fundamental frequency data (*n* = 1) are usually disregarded due to sensitivity to environmental noise,^24^ but we included the data to monitor the process of macroscopic film removal. All experiments were conducted at 25 °C.

### Spectroscopic Ellipsometer (SE) Measurements

The thickness of the “thin photoresist film” in air was measured by using an SE instrument (FS-1, Film Sense). A substrate coated with the photoresist was exposed to wavelengths of 465 nm (blue), 525 nm (green), 580 nm (yellow), and 635 nm (red) at an incident angle of 65°. In this study, a silicon wafer was chosen as the substrate beneath the photoresist for simplified analysis. The algorithm used to determine the thickness aimed to minimize the difference between the experimental data and the optical model for all wavelengths.

### CLSM Observations

The “thick photoresist films” were observed using CLSM (LSM 800, Carl ZEISS). The fluorophores Rhodamine 110 chloride and Rhodamine B were excited using diode lasers with 488 and 561 nm, and fluorescence was measured in the range of 498–530 nm (green) and 571–650 nm (red), respectively.^11^ Fluorescence was recorded by employing a GaAsP detector, and the obtained data were visualized in two dimensions (2D) and three dimensions (3D) using Imaris software.

Rhodamine 110 chloride was added directly to the removal agent, while Rhodamine B was added to the PGMEA/photoresist solution prior to the coating at approximately 50 μmol L^–1^ each. The photoresist films were formed according to the “photoresist film formation” section. The Rhodamine 110 chloride-stained solution (volume: 20 μL) was dropped onto the Rhodamine B-stained photoresist on the ITO substrate, and the state was observed after 10 min.

### Threshold Analysis

Overhead visual images of the photoresist coatings were thresholded by using ImageJ software. For analysis, time-resolved images of the “thick photoresist film” were obtained with the removal agent. After converting the image to an 8-bit grayscale, binarization was performed to separate the shades between the photoresist and the substrate, and the number, area, and size of the photoresist particles were quantified.

## Results and Discussion

### Photoresist Film Characterization

We characterized the dried photoresist film on an ITO substrate. The photoresist was coated on the QCM-D ITO sensor (3000 rpm, 30 s), resulting in a visually uniform and reddish appearance (Figure S1). Subsequently, the water contact angle increased from 17.7° on the bare ITO sensor to 81.9° on the photoresist film (Figure S1), indicating that the photoresist (novolak resin) imparted hydrophobicity to the ITO surface. These contact angles closely matched the reported values.^25−27^ The mass and thickness of the “thin photoresist film” were estimated using QCM-D (ITO-coated sensor) and SE (silica) techniques, respectively. The change in the resonance frequency before and after film formation was calculated by QCM-D, yielding the film mass (Table S1). The Sauerbrey equation (eq 1)^22^ was utilized to quantify the film mass of approximately 630 mg m^–2^, and the SE measurement on silica determined the photoresist film thickness to be approximately 450 nm (the optical constants are provided in Table S2). Additionally, we ensured that the fundamental energy dissipation difference in the QCM-D measurements remained below 0.5 × 10^–6^. The photoresist density was calculated as 1.3 × 10^3^ kg m^–3^ using the obtained QCM-D mass and SE thickness, which agreed well with the literature value of 1.25 × 10^3^ kg m^–3^.^28^

In contrast, visual examination of the “thick photoresist film” (1000 rpm, 10 s) revealed a thickness of 10 μm, as shown in the CLSM image (Figure S2). This difference in thickness arose due to the dependence of photoresist film mass and thickness on the coating rotation speed and time.^29^

### Interaction between the Photoresist Film and Solvent without the Pluronic Surfactant

**Figure 1:**
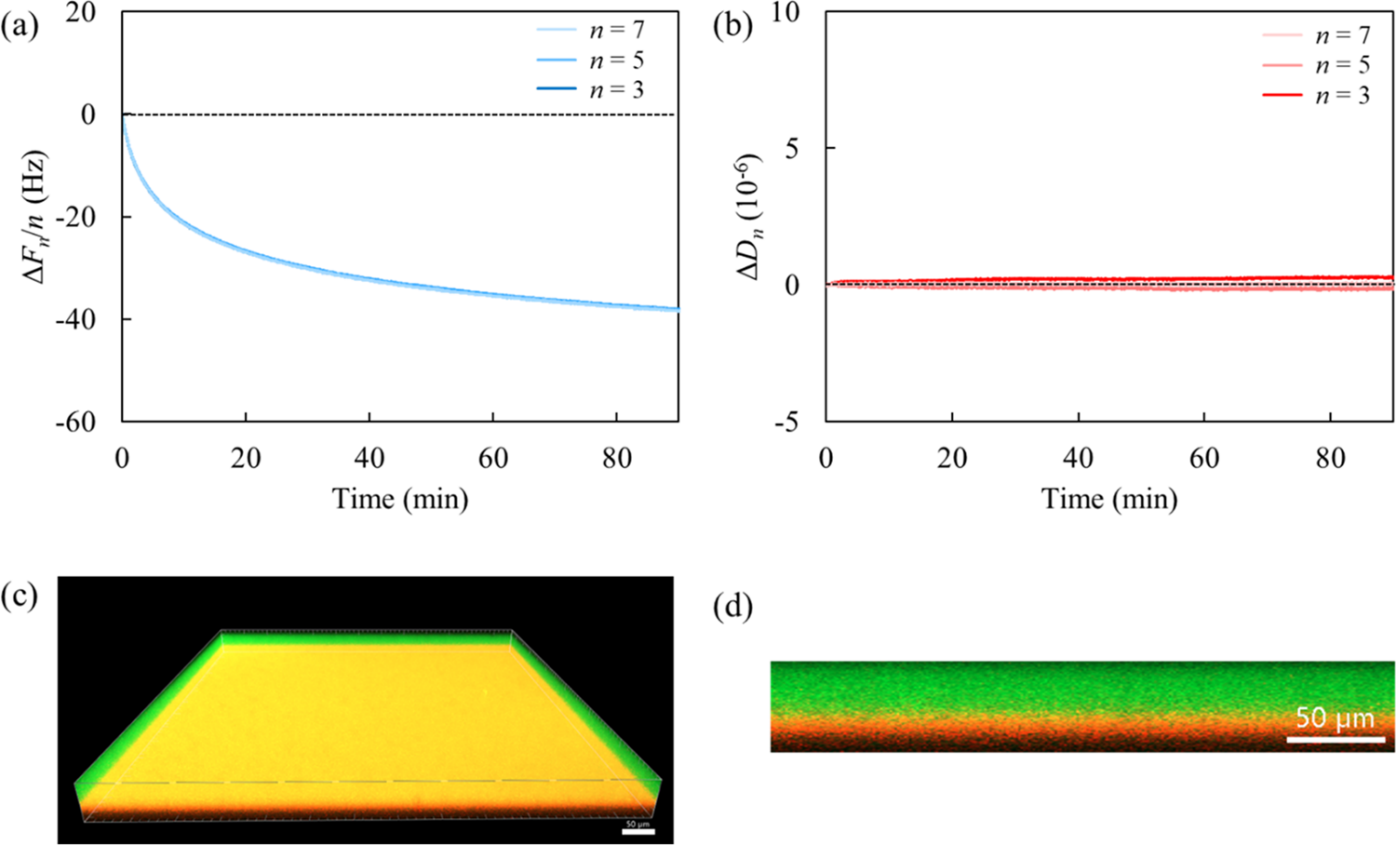
(a) Frequency and (b) dissipation shifts as a function of time of the third, fifth, and seventh overtones in pure water measured for the photoresist film coated on the ITO substrate. CLSM images of the Rhodamine-B-labeled photoresist film (red) in Rhodamine-110-chloride-labeled water (green): (c) 3D overview and (d) 2D cross-section representations. The scale bar corresponds to 50 μm in length.

We examined the interaction between a “thin photoresist film” and each agent without the Pluronic surfactant. Figure 1a,b shows the QCM-D results during the exposure of the photoresist film on the ITO-sensor to pure water. Before the injection of pure water, the Δ*F*_*n*_/*n* and Δ*D*_*n*_ were stabilized in air for all overtones. To exclude shifts caused by variations in viscosity and density (the bulk effect) between air and water, the time “0 min” was assigned to a state in which the module was completely filled with water. Δ*F*_*n*_/*n* decreased to approximately −40 Hz at 90 min, whereas Δ*D*_*n*_ remained stable near the baseline level. The decrease in Δ*F*_*n*_/*n* in Figure 1a indicates a slight uptake of water into the film with stable viscoelasticity.

A comparison between overtones provides information about the penetration depth of shear waves in medium.^23,30^ When the viscoelasticity and thickness of the film on the sensor remain almost constant, the overtone data typically exhibit the same profile, as displayed in Figure 1a,b. In the case of Paraloid B72 (acrylic resin) on a gold sensor, water penetrating into the film affects the viscoelasticity of the entire acrylic film, resulting in overtone-dependent frequency and dissipation shifts.^11^ Thus, photoresists (novolak resin) are likely to be more resistant to water than Paraloid B72 because of their different chemical structures (polar/nonpolar balance and molecular weight).

Figure 1c,d shows the CLSM images of the “thick photoresist film” in water. A photoresist with a low affinity for water retains its morphology on the ITO substrate without erosion from the liquid phase. This repellency indicates that water, a poor solvent for the photoresist, does not penetrate inside the film and interacts only with the surface of the film. Conversely, we did not observe leakage of Rhodamine B into the liquid phase because of its low partition coefficient (log *P* = approximately 1.8)^31^ and its immobilization in the film.

Before using a mixed solvent of EC/PC and water, we confirmed the bulk effects associated with viscosity and density changes between mediums in QCM-D measurements.^32^ The measured QCM-D responses are given in Figure S3. As the amount of EC/PC in the solvent increased, the shifts from the baseline level (pure water) in Δ*F*_*n*_/*n* and Δ*D*_*n*_ increased. This bulk effect can be a measure of the contribution of the solution to the mass and viscoelasticity of the coating film.

**Figure 2:**
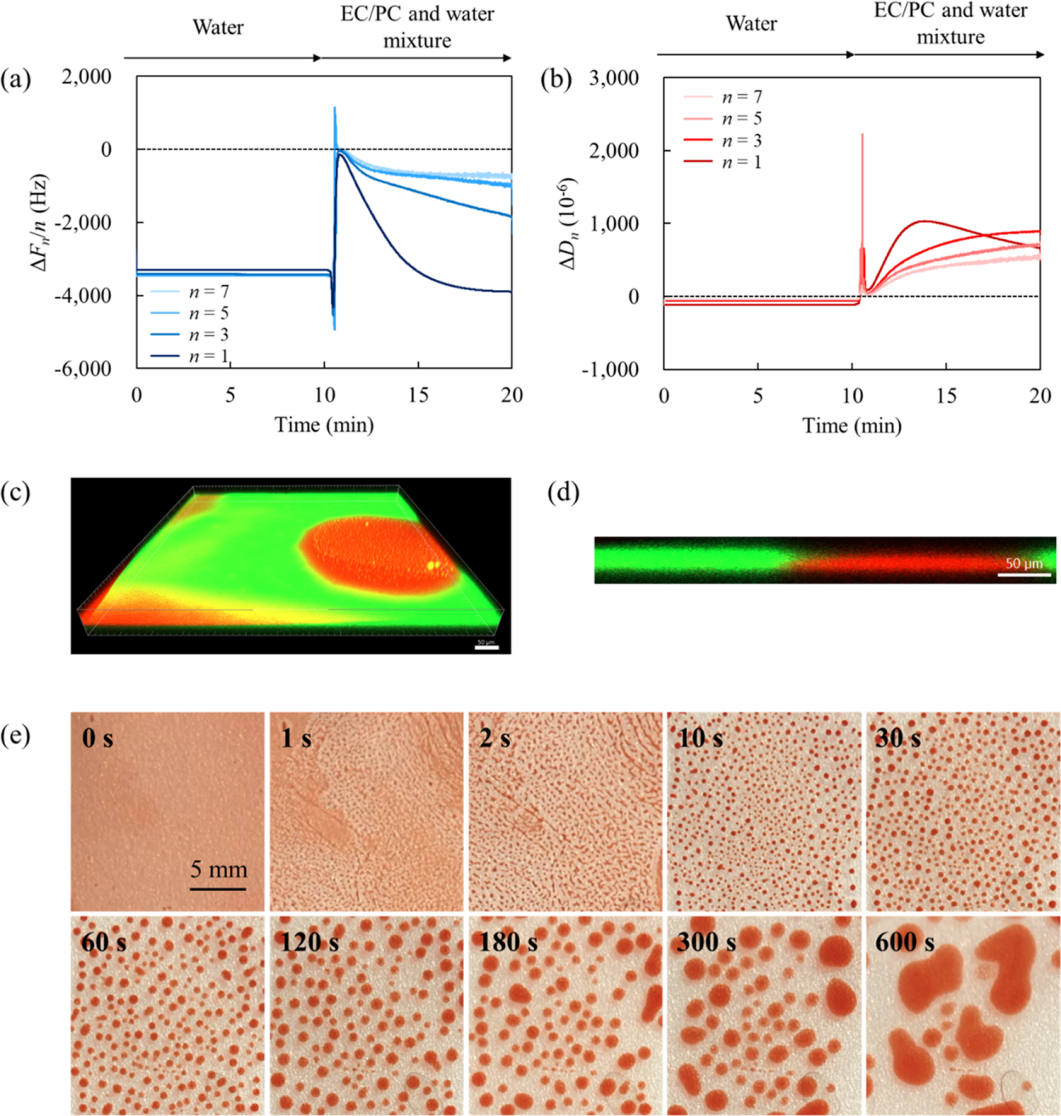
(a) Frequency and (b) dissipation shifts as a function of time of the first, third, fifth, and seventh overtones for the interaction between the photoresist film and EC/PC-to-water mixture. CLSM images of the Rhodamine-B–labeled photoresist film (red) in Rhodamine-110-chloride–labeled EC/PC-to-water mixture (green): (c) 3D overview and (d) 2D cross-sectional representations. The scale bar in the CLSM images corresponds to 50 μm in length. (e) Time-resolved visual representations of the photoresist film in the EC/PC-to-water mixture.

Figure 2a,b shows the QCM-D results when a “thin photoresist film” on the ITO-sensor was exposed from pure water to the EC/PC-to-water mixture (the weight ratio was set at 50/50). Here, the baseline level corresponds to bare ITO in the EC/PC-to-water mixture; therefore, the photoresist film reaches the baseline upon complete desorption. The complex profiles of Δ*F*_*n*_/*n* and Δ*D*_*n*_ shown in Figure 2a,b are ascribed to a three-step process: (i) precipitous Δ*F*_*n*_/*n* drop above 1000 Hz; (ii) reaching Δ*F*_*n*_/*n* near the baseline level; and (iii) gradual Δ*F*_*n*_/*n* decrease with overtone divergence; Δ*D*_*n*_ behaved according to the Δ*F*_*n*_/*n* profile. In the first step, the shifts of Δ*F*_*n*_/*n* and Δ*D*_*n*_ are greater than the bulk effect (Figure S3), indicating that EC and PC molecules, which are good solvents for the photoresist, penetrated into the film and caused subsequent swelling. Then, we assumed the detachment of the photoresist film from the substrate (the second step); however, the photoresist unexpectedly readsorbed with softening (the third step).

Figure 2c,d presents the CLSM images of a “thick photoresist film” in contact with the EC/PC-to-water mixture for 10 min. Here, it can be observed that the red-stained photoresist adopts a hemispherical morphology, unlike that in pure water (Figure 1c,d). Moreover, the interior of the hemisphere in Figure 2c is dotted with a green-stained solution. A photoresist film with a thickness equivalent to that of the CLSM analysis was submerged in the EC/PC-to-water mixture (Figure 2e). Within 2 s, fine particles began forming on the film surface, and within 10 s, nearly all of them exhibited a spherical shape. The dewetting process was driven by the presence of EC and PC in the solvent; nevertheless, the size of the photoresist particles continued to increase over time.

**Figure 3:**
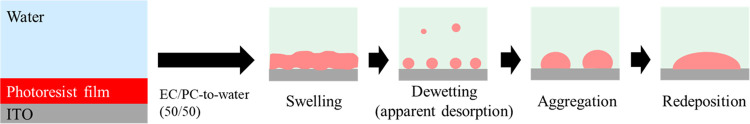
Schematic representation of the interaction between the photoresist film and EC/PC-to-water mixture without Pluronic surfactant on the ITO substrate.

Note that the photoresist film thickness differs across measurements; however, the QCM-D results align with CLSM and visual observations. The instantaneous swelling of the film in the first QCM-D step corresponds to approximately 1–2 s. This process confirms the significance of softening the rigid photoresist prior to deformation. Subsequently, the return to nearly baseline levels of frequency and energy dissipation shifts (the second QCM-D step) signifies the formation of grains on the ITO through dewetting, rather than complete removal from the substrate.^33^ We hypothesize that the very small contact area between the particles and the substrate weakens the response to shear waves, leading to the apparent desorption. In particular, the frequencies at high overtones (25 and 35 MHz) exhibited positive shifts. This is presumably due to the resonance frequency of the resultant photoresist particles being lower than that of the sensor.^34,35^ The third step of continuous redeposition reflects the coalescence of particles on the ITO substrate. The separation between overtone profiles (greater shifts for lower overtones) is consistent with the softening and thickening of entire photoresist particles through coalescence among dewetted particles. In the absence of Pluronic surfactants, the photoresist films dewetted and aggregated on the ITO substrate, as schematically shown in Figure 3.

### Effect of Solvent–Polymer Affinity on Dewetting

We applied Hansen solubility parameters (HSPs)^36,37^ to evaluate the miscibility between the solvent and photoresist. This association enables the prediction of dewetting promotion and has the potential to be a tool for the design of removal agents. The HSP is composed of three contributions from dispersion (δ_d_), polar (δ_p_), and hydrogen bonding (δ_h_) components, as defined in eq 4,
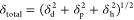
4where δ_total_ is the total (Hildebrand) solubility parameter. The miscibility between the solvent and the polymer is compared in terms of *R*_a_ (eq 5); a lower *R*_a_ indicates a greater miscibility between materials 1 and 2.
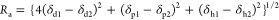
5The relative energy difference (RED), defined by the ratio of *R*_a_ to radius *R*_0_ of the interaction sphere, is represented by eq 6, as follows.
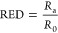
6

When RED is less than 1, the solvent is a good solvent for the polymer, while when RED is more than 1, the solvent is a poor solvent for the polymer.

Before discussing HSPs in this study, we demonstrated the validity of HSPs using phenolic resin, which is the primary component of the photoresist. The visual representations of the photoresist reported in Figure S4 reveal the insolubility or sparing solubility in low-polarity solvents such as hexane, toluene, and chloroform. In contrast, it was confirmed that the photoresist dissolves in high-polarity solvents. Table S3 shows the HSPs of the phenolic resin and each corresponding material in Figure S4. The calculated RED values with phenolic resin are clearly consistent with the solubility test, indicating that phenolic resin will be a suitable alternative for the HSP of the photoresist.

In practice, we calculated HSP, *R*_a_, and RED of EC/PC, EC/PC-to-water mixture, and water with phenolic resin; these values are listed in Table 1. For the HSP calculation of the mixed solvent (δ_mix_), eq 7 was derived based on the principle that additivity is established in the volume ratio,^38^
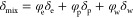
7where φ and δ are the volume fraction and solubility parameter and subscripts e, p, and w present EC, PC, and water, respectively. The *RED* values are in agreement with its visual state (Figure S5) as well as the relationship between Table S3 and Figure S4. Because of the immiscibility between water and the photoresist, the RED value exceeds 1 for at least 50% or more of the volume of water in the solvent. This trend is also observed in the QCM-D results (Figures 1 and 2). Specifically, in the EC/PC-poor solution (Figure S6), only the solvents penetrate the photoresist film without deformation, while in the EC/PC-rich solution (Figure S7), the film detaches from the ITO sensor without swelling. This promotes dewetting through solvent penetration and film deformation within a limited range of RED values, as shown in Figure 2.

**Table 1:** HSP and Calculated RED Values of Each Material

materials	δ__total__ [MPa^^1/2^^]	δ__h__ [MPa^^1/2^^]	δ__p__ [MPa^^1/2^^]	δ__d__ [MPa^^1/2^^]	*R*__0__ [MPa^^1/2^^]	*R*__a__ [MPa^^1/2^^]	RED
phenolic resin	27.1	14.6^a^	11.6^a^	19.7^a^	12.7^a^		
EC/PC	31.0	11.8^b^	20.8^b^	19.6^b^		9.6	0.76
EC/PC-to-water (75/25)	34.0	21.2	19.3	18.4		10.5	0.83
EC/PC-to-water (50/50)	38.4	29.2	18.1	17.3		16.7	1.3
EC/PC-to-water (25/75)	43.2	36.2	17.0	16.3		23.2	1.8
Water	47.8	42.3^a^	16.0^a^	15.5^a^		29.3	2.3

a From ref (42).

b From ref (43).

Dewetting of polymer films on a solid surface is influenced by the interfacial free energy as well as the mobility of polymer chains.^39,40^ The balance between wetting and dewetting is characterized by the spreading coefficient, *S*, which is defined in eq 8 based on Young’s equation,^40,41^
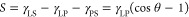
8where γ is the interfacial tension (liquid-L, solid-S, and polymer-P). The γ_PS_ is constant in this study, and the γ_LS_ is almost identical for EC/PC and water, both of which are highly polar,^21^ while the γ_LP_ depends on the solvent polarity (poor-solvent water has a high γ_LP_ value; good-solvent EC/PC has a low γ_LP_ value). Theoretically, the film dewetting should be promoted, and the contact angle should increase as the amount of water content increases. This requires a consideration of the dynamics of the polymer chain mobility. Given the premise that *S* becomes negative, the photoresist film dewetting drives on the substrate within a limited range of RED of more than 1.

### Interaction between the Photoresist Film and Solution with the Pluronic Surfactant

**Figure 4:**
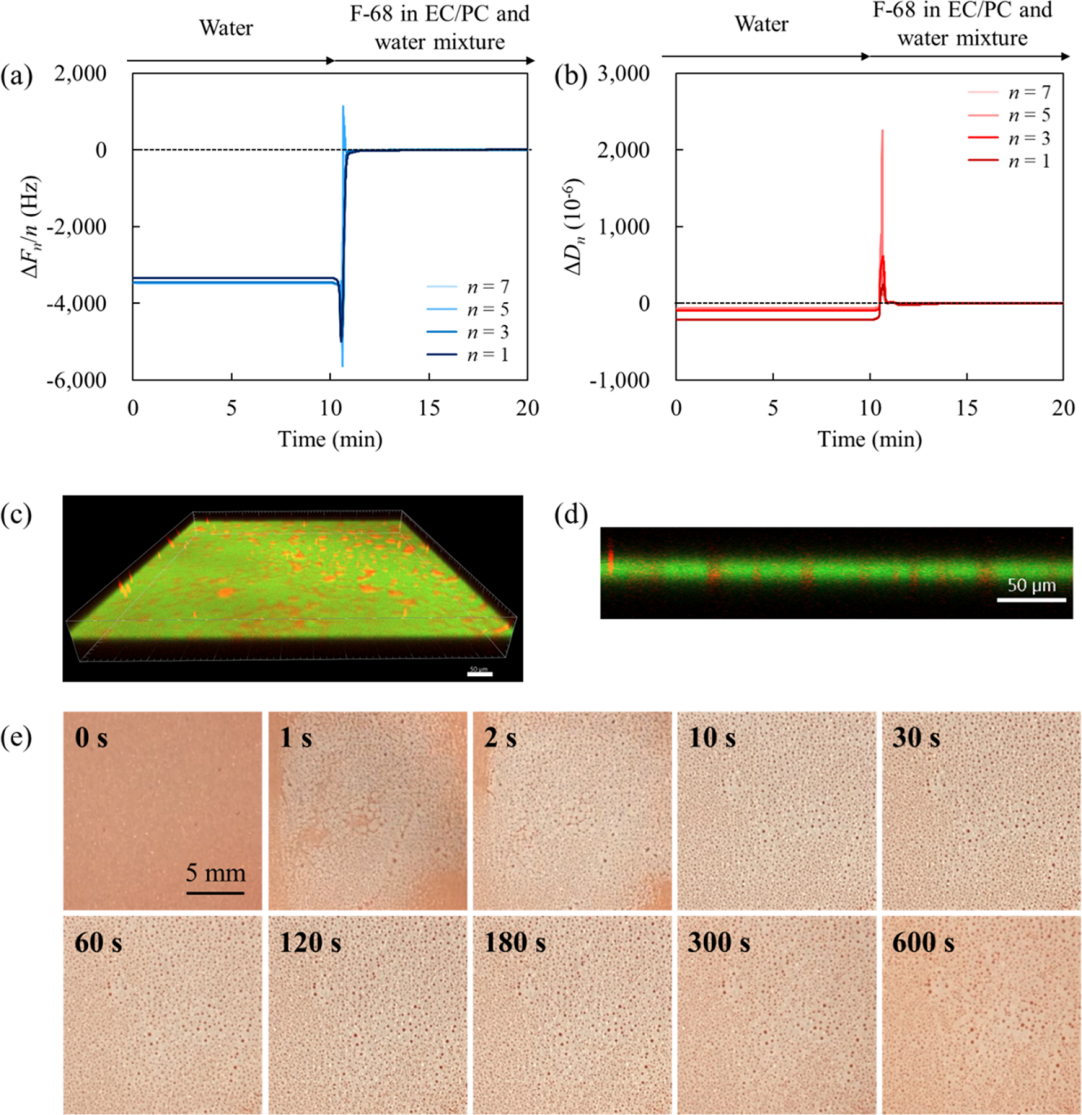
(a) Frequency and (b) dissipation shifts as a function of time of the first, third, fifth, and seventh overtones for the interaction between the photoresist film and EC/PC-to-water mixture with F-68 (1% w/w). CLSM images of the Rhodamine-B-labeled photoresist film (red) in Rhodamine-110-chloride-labeled EC/PC-to-water mixture with F-68 (green): (c) 3D overview and (d) 2D cross-sectional representations. The scale bar in the CLSM images corresponds to 50 μm in length. (e) Time-resolved visual representations of the photoresist film in the EC/PC-to-water mixture.

Figure 4a,b shows the QCM-D results obtained in the presence of Pluronic F-68. The QCM-D measurement was performed under the same conditions as the injection procedure, baseline definition, and solvent mixed ratio shown in Figure 2a,b. When the F-68 solution was replaced with pure water on a “thin photoresist film”, Δ*F*_*n*_/*n* decreased by the range of 1600 (*n* = 1)–2200 (*n* = 7) Hz, followed by an immediate increase near the baseline level regardless of overtones. These shifts, as for Δ*D*_*n*_, are caused by changes in film properties that are much greater than bulk effects and also very similar to the profiles up to the “first” and “second” steps in the system without F-68 (Figure 2a,b). However, the frequency and dissipation shifts stabilized on the baseline in the presence of F-68 without proceeding to the “third” step with redeposition of the photoresist. This indicated that F-68 suppressed the aggregation of the photoresist and promoted the detachment of the film from the ITO surface.

The CLSM images depicted in Figure 4c,d exhibit noteworthy morphological variations, including film subdivision, solvent penetration, and lift-up from the substrate, when compared to the absence of F-68 as shown in Figure 2c,d. Figure 4e presents time-resolved visual observation results for the photoresist film under the same conditions as for CLSM. Initially, the film dewetted within the first few seconds and maintained its spherical morphology after 10 s; this corresponds to the steady state observed in the QCM-D results. The disparity in photoresist residue magnitude between the QCM-D results and observation data can be influenced by film thickness and external force (i.e., continuous solution flow during QCM-D measurements). To provide additional information, we performed substrate shaking after immersing a “thick photoresist film” for 10 min (visual representations are shown in Figure S8). Even after shaking, the photoresist remained firmly attached to the substrate without the presence of F-68. In contrast, with F-68, the photoresist easily detached and dispersed into the bulk solution, supporting a series of experimental findings.

**Figure 5:**
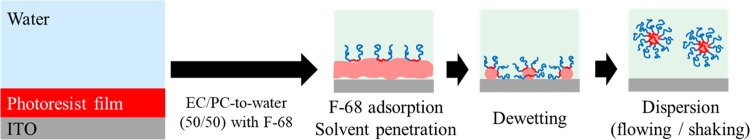
Schematic representation of the interaction between the photoresist film and the EC/PC-to-water mixture with F-68 on the ITO substrate.

We calculated the removal efficiency of the photoresist film from the frequency change before and after the QCM-D cleaning test of the photoresist-coated ITO sensor, as per a previous study.^44^ The removal efficiency was 66.0% without F-68 and 99.8% with F-68, indicating that the addition of F-68 imparts an effect that cannot be elucidated by HSP theory. In the presence of Pluronic F-68, the photoresist films are removed from the ITO substrate, as schematically shown in Figure 5.

### Effect of Pluronic Surfactants on Photoresist Dewetting

**Figure 6:**
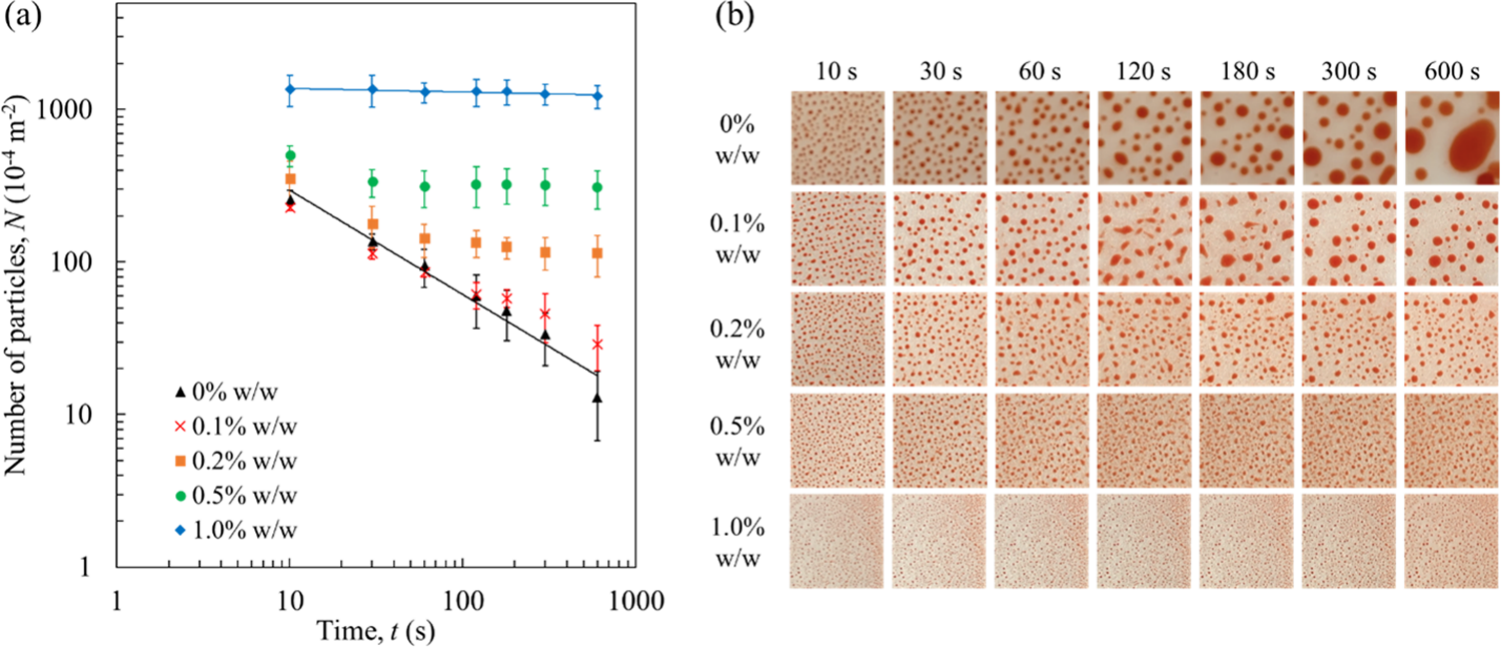
(a) Number of the photoresist particles calculated from (b) their visual observations of the photoresist films immersed in the EC/PC-to-water mixture without and with F-68 as a function of time (standard deviation intervals; *N* = 3). The weight ratio of EC/PC and water was fixed at 50/50; the F-68 concentrations added to the solvent were set at 0, 0.1, 0.2, 0.5, and 1.0% w/w. The solid lines represent fittings based on power law decay corresponding to the experimental data. All images in (b) are represented as 1 cm × 1 cm square.

Figure 6a depicts the relationship between the time and the number of photoresist particles formed through dewetting. This image analysis was conducted from 10 s onward, when the particles were fully formed. To understand the dewetting mechanism of the photoresist, we included data for different F-68 concentrations in the graph (visual representations corresponding to these data are shown in Figure 6b). The number of photoresist particles on the ITO substrate decreased over time, with the extent of decrease depending on the F-68 concentration in the solvent. As F-68 forms micelles at or above 40% w/w in water at 25 °C,^45^ these profiles are expected to be contingent on the concentration of F-68 unimers. However, in certain systems, the declining trend in particle counts reached a plateau at intermediate time points (from 30 s onward at 0.5% w/w; from 200–300 s onward at 0.2% w/w). According to the Langmuir kinetic model, the reduction in F-68 molecules leads to a decrease in diffusion from the bulk solution to the solid surface, thereby lowering the adsorption rate on the photoresist surface.^46,47^ The number of particles may be regulated based on the balance of interaction between the repulsive force of the F-68 adsorption layer^15,21^ and the attractive force originating from the photoresist.

Figure 6a also shows power law decay profiles (*N* = *At*^–*x*^) for the experimental data with linearity in the absence and presence of F-68. The data were fitted by applying linear least-squares to the log-transformed graphs; the coefficient of determination, *R*^2^, was found to be 0.97 and 0.83 without and with F-68 (1% w/w), respectively. As indicated by the obtained fitted parameters (*A* = 1433 and *x* = −0.684 in the absence of F-68; *A* = 1454 and *x* = −0.024 in the presence of F-68 1% w/w), F-68 affected coefficient *x*, which is the factor of particle aggregation rate, as opposed to coefficient *A* (the factor of initial number of the particles).

**Figure 7:**
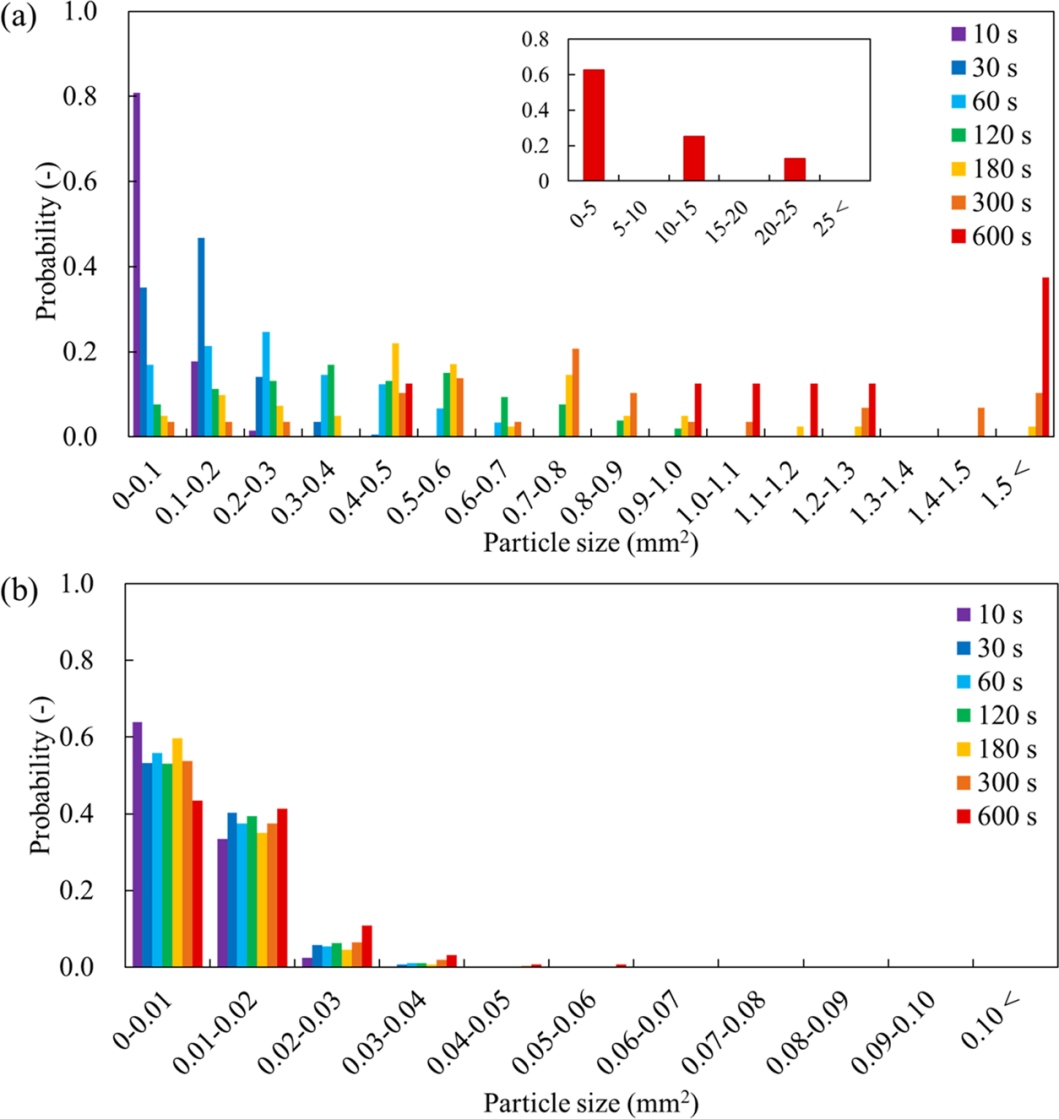
Time-dependent size histograms of the photoresist particles obtained in the EC/PC-to-water mixture (a) without and (b) with F-68 (1% w/w) on the ITO substrate.

Figure 7 shows the time-dependent size histograms of the photoresist particles. In the absence of F-68, the majority of photoresist particles was within 0.1 mm^2^ at 10 s, but the particle size increased, and broadening of the size distribution occurred over time. This indicates that the photoresist particles coalesced randomly with adjacent particles on the ITO substrate, resulting in an increased occupied area of each particle (Figure S9). In contrast, the particle size and occupied area were almost unchanged in the presence of F-68. According to the consistent trends in number, size, and occupied area of the photoresist particles, F-68 exerts an anticoalescing effect by stabilizing photoresist particles.

Thus, we hypothesize that F-68 contributes to the kinetics rather than the thermodynamics of the dewetting associated with polymer chain mobility. A decrease in γ_LP_ with F-68 adsorption on the photoresist leads to convergence of the spreading coefficient, *S*, to zero and stabilizes the photoresist film (eq 8). Under solvent conditions that cause only swelling without dewetting, F-68 not only swells the photoresist film but also retains the elasticity of the film (remarkably low Δ*D*_*n*_ behavior for the F-68 system is shown in Figure S10). Hence, F-68 impedes the increase in the polymer mobility (i.e., softening the film) that thermodynamically favors dewetting. This stabilization mechanism caused by F-68 adsorption is more effective in the process of increasing the interfacial regions between the liquid–solid and liquid–polymer during dewetting. As described earlier, the diffusion rate of F-68 unimers is essential for the kinetics of photoresist growth via dewetting. The kinetically competitive interaction (photoresist aggregation with high interfacial free energy and adsorption by diffusion of F-68) plays a significant role in the removal mechanism of the photoresist in the dewetting process, driven by a mixture of poor and good solvents with a limited RED value.

## Conclusions

In this article, we reported on the ability of Pluronic F-68 to remove a photoresist coating from an ITO substrate in a mixture of good solvents (EC and PC) and a poor solvent (water). CLSM measurements demonstrated that in the mixed solvent of EC/PC and water, the photoresist dewetted and changed from a flat shape to a spherical shape. QCM-D measurements revealed that the photoresist film dewets via several processes. First, the photoresist film swells by the penetration of good solvents. Then, the softened photoresist dewets on the substrate due to the presence of water. Subsequently, the photoresist particles gradually aggregate on the substrate, which is consistent with HSP calculations showing the insolubility between the solvent and the novolak resin. The aggregation-induced increase in contact area between the photoresist particles and the substrate was reflected in the shifts as decreased frequency and increased energy dissipation in the QCM-D measurements. In contrast, Pluronic F-68 in the EC/PC-to-water mixture inhibited the aggregation of the dewetted particles, resulting in promoted desorption of the photoresist from the substrate. This series of behaviors is monitored up to complete removal from the substrate within a minute and is supported by CLSM analysis, which observed the morphological changes of the photoresist coating, such as subdivision of the film, solvent penetration in the film, and lift-up from the substrate. The number of photoresist particles was kinetically controlled by the photoresist aggregation rate and adsorption rate due to the diffusion of F-68 molecules.

This proposed removal mechanism using three components—good solvents, poor solvents, and Pluronic surfactants—provides the possibility of achieving both low environmental impact and high cleaning performance in the electronics industry. This formulation has the potential to provide a solution for wide varieties of industries with these trade-off issues. Furthermore, combining interfacial analysis tools with HSP calculations will contribute to the development of chemical cleaning agents from multiple perspectives. Future studies will focus on different combinations of solvents and surfactants to find a recipe for photoresist removal. Additional strategies will also be required for cleaning micropatterns of electronic devices, which may increase the versatility of QCM-D measurements by using flat substrates.

## Supporting Information Available

Characterization of the photoresist film, bulk effect in QCM-D measurements, validation of HSP values for the photoresist, removal of photoresist films without Pluronics, visual representation of photoresist films, analysis of photoresist particles using ImageJ, and stabilization of photoresist films with F-68 (PDF)

## Abbreviations

2D: two dimensions
3D: three dimensions
CLSM: confocal laser scanning microscopy
EC: ethylene carbonate
HSP: Hansen solubility parameter
ITO: indium tin oxide
PC: propylene carbonate
PEO: poly(ethylene oxide)
PGMEA: propylene glycol monomethylethyl ether acetate
PPO: poly(propylene oxide)
QCM-D: quartz crystal microbalance with dissipation monitoring
RED: relative energy difference
SE: spectroscopic ellipsometer
UV: ultraviolet

## References

[ref1] Rosan M. J.; Kunjappu J. T. Surfactants and Interfacial Phenomena, 4th ed.; Wiley, 2012; pp 392–420.

[ref2] Bajpai D.; Tyagi V. K. Laundry Detergents: An Overview. J. Oleo Sci. 2007, 56, 327–340. 10.5650/jos.56.327. 17898499

[ref3] Gecol H.; Scamehorn J. F.; Christian S. D.; Grady B. P.; Riddell F. Use of Surfactants to Remove Water Based Inks from Plastic Films. Colloids Surf., A 2001, 189, 55–64. 10.1016/S0927-7757(01)00591-X.

[ref4] Rebello S.; Asok A. K.; Mundayoor S.; Jisha M. S. Surfactants: Toxicity, Remediation and Green Surfactants. Environ. Chem. Lett. 2014, 12, 275–287. 10.1007/s10311-014-0466-2.

[ref5] Yan J.; Dhane K.; Vermeire B.; Shadman F. In-situ and Real-Time Metrology During Rinsing of Micro- and Nano-Structures. Microelectron. Eng. 2009, 86, 199–205. 10.1016/j.mee.2008.10.014.

[ref6] Baglioni P.; Carretti E.; Chelazzi D. Nanomaterials in Art Conservation. Nat. Nanotechnol. 2015, 10, 287–290. 10.1038/nnano.2015.38. 25855252

[ref7] Wei Q.; Becherer T.; Angioletti-Uberti S.; Dzubiella J.; Wischke C.; Neffe A. T.; Lendlein A.; Ballauff M.; Haag R. Protein Interactions with Polymer Coatings and Biomaterials. Angew. Chem., Int. Ed. 2014, 53, 8004–8031. 10.1002/anie.201400546. 25045074

[ref8] Deshpande P. P.; Jadhav N. G.; Gelling V. J.; Sazou D. Conducting Polymers for Corrosion Protection: A Review. J. Coat. Technol. Res. 2014, 11, 473–494. 10.1007/s11998-014-9586-7.

[ref9] Lu T.; Reimonn G.; Morose G.; Yu E.; Chen W. T. Removing Acrylic Conformal Coating with Safer Solvents for Re-Manufacturing Electronics. Polymers 2021, 13, 937 10.3390/polym13060937. 33803712 PMC8002995

[ref10] Raudino M.; Selvolini G.; Montis C.; Baglioni M.; Bonini M.; Berti D.; Baglioni P. Polymer Films Removed from Solid Surfaces by Nanostructured Fluids: Microscopic Mechanism and Implications for the Conservation of Cultural Heritage. ACS Appl. Mater. Interfaces 2015, 7, 6244–6253. 10.1021/acsami.5b00534. 25723546

[ref11] Raudino M.; Giamblanco N.; Montis C.; Berti D.; Marletta G.; Baglioni P. Probing the Cleaning of Polymeric Coatings by Nanostructured Fluids: A QCM-D Study. Langmuir 2017, 33, 5675–5684. 10.1021/acs.langmuir.7b00968. 28537736

[ref12] Soyano A. Application of Polymers to Photoresist Materials. Int. Polym. Sci. Technol. 2012, 39, 47–54. 10.1177/0307174X1203900513.

[ref13] Ota H.; Otsubo H.; Yanagi M.; Fujii H.; Kamimoto Y. A New Eco-friendly Photo Resist Stripping Technology Using “Ethylene Carbonate. IEICE Trans. Electron. 2010, E93–C, 1607–1611. 10.1587/transele.E93.C.1607.

[ref14] Shaikh A. A.; Sivaram S. Organic Carbonates. Chem. Rev. 1996, 96, 951–976. 10.1021/cr950067i. 11848777

[ref15] Hanzawa M.; Ogura T.; Tsuchiya K.; Akamatsu M.; Sakai K.; Sakai H. Anti-adsorption Mechanism of Photoresist by Pluronic Surfactants: An Insight into Their Adsorbed Structure. Langmuir 2023, 39, 7876–7883. 10.1021/acs.langmuir.3c00714. 37209170 PMC10249399

[ref16] Marx K. A. Quartz Crystal Microbalance: A Useful Tool for Studying Thin Polymer Films and Complex Biomolecular Systems at the Solution–Surface Interface. Biomacromolecules 2003, 4, 1099–1120. 10.1021/bm020116i. 12959572

[ref17] Sadman K.; Wiener C. G.; Weiss R. A.; White C. C.; Shull K. R.; Vogt B. D. Quantitative Rheometry of Thin Soft Materials Using the Quartz Crystal Microbalance with Dissipation. Anal. Chem. 2018, 90, 4079–4088. 10.1021/acs.analchem.7b05423. 29473414

[ref18] Kaga H.; Nakamura A.; Orita M.; Endo K.; Akamatsu M.; Sakai K.; Sakai H. Removal of a Model Biofilm by Sophorolipid Solutions: A QCM-D Study. J. Oleo Sci. 2022, 71, 663–670. 10.5650/jos.ess21360. 35387914

[ref19] Mohona T. M.; Dai N.; Nalam P. C. Comparative Degradation Kinetics Study of Polyamide Thin Films in Aqueous Solutions of Chlorine and Peracetic Acid Using Quartz Crystal Microbalance. Langmuir 2021, 37, 14214–14227. 10.1021/acs.langmuir.1c02835. 34793175

[ref20] Olesen K.; van Leeuwen C.; Andersson F. I. Revealing Detergent Efficiency and Mechanism by Real-Time Measurement Using a Novel and Tailored QCM-D Methodology. Tenside, Surfactants, Deterg. 2016, 53, 488–494. 10.3139/113.110445.

[ref21] Hanzawa M.; Oohinata H.; Kawano S.; Akamatsu M.; Sakai K.; Sakai H. Adsorption of Pluronic Surfactants in Alkylene Carbonates on Silica. Langmuir 2018, 34, 14180–14185. 10.1021/acs.langmuir.8b02543. 30404452

[ref22] Sauerbrey G.; von Schwingquarzenzur V. W. Igung Diinner Schichten und zur Mikrowaigung. Z. Phys. 1959, 155, 206–222. 10.1007/BF01337937.

[ref23] Voinova M. V.; Rodahl M.; Jonson M.; Kasemo B. Viscoelastic Acoustic Response of Layered Polymer Films at Fluid-Solid Interfaces: Continuum Mechanics Approach. Phys. Scr. 1999, 59, 391–396. 10.1238/Physica.Regular.059a00391.

[ref24] Dutta A. K.; Belfort G. Adsorbed Gels versus Brushes: Viscoelastic Differences. Langmuir 2007, 23, 3088–3094. 10.1021/la0624743. 17286418 PMC3953464

[ref25] Morita M.; Ohmi T.; Hasegawa E.; Kawakami M.; Ohwada M. Growth of Native Oxide on a Silicon Surface. J. Appl. Phys. 1990, 68, 1272–1281. 10.1063/1.347181.

[ref26] So S. K.; Choi W. K.; Cheng C. H.; Leung L. M.; Kwong C. F. Surface Preparation and Characterization of Indium Tin Oxide Substrates for Organic Electroluminescent Devices. Appl. Phys. A: Mater. Sci. Process. 1999, 68, 447–450. 10.1007/s003390050921.

[ref27] Kamal T.; Hess D. W. Photoresist Removal Using Low Molecular Weight Alcohols. J. Electrochem. Soc. 2000, 147, 2749–2753. 10.1149/1.1393600.

[ref28] Shibayama M.; Shudo Y.; Izum A. Structure and Functions of Phenolic Resin. J. Adhes. Soc. Jpn. 2018, 54, 451–458. 10.11618/adhesion.54.451.

[ref29] Meyerhofer D. Characteristics of Resist Films Produced by Spinning. J. Appl. Phys. 1978, 49, 3993–3997. 10.1063/1.325357.

[ref30] Dunér G.; Thormann E.; Dėdinaitė A. Quartz Crystal Microbalance with Dissipation (QCM-D) Studies of the Viscoelastic Response from a Continuously Growing Grafted Polyelectrolyte Layer. J. Colloid Interface Sci. 2013, 408, 229–234. 10.1016/j.jcis.2013.07.008. 23932084

[ref31] Kojic M.; Milosevic M.; Wu S.; Blanco E.; Ferrari M.; Ziemys A. Mass Partitioning Effects in Diffusion Transport. Phys. Chem. Chem. Phys. 2015, 17, 20630–20635. 10.1039/C5CP02720A. 26204522 PMC4527522

[ref32] Voinova M. V.; Jonson M.; Kasemo B. ‘Missing Mass’ Effect in Biosensor’s QCM Applications. Biosens. Bioelectron. 2002, 17, 835–841. 10.1016/S0956-5663(02)00050-7. 12243901

[ref33] Vayer M.; Vital A.; Sinturel C. New Insights into Polymer-Solvent Affinity in Thin Films. Eur. Polym. J. 2017, 93, 132–139. 10.1016/j.eurpolymj.2017.05.035.

[ref34] Pomorska A.; Shchukin D.; Hammond R.; Cooper M. A.; Grundmeier G.; Johannsmann D. Positive Frequency Shifts Observed Upon Adsorbing Micron-Sized Solid Objects to a Quartz Crystal Microbalance from the Liquid Phase. Anal. Chem. 2010, 82, 2237–2242. 10.1021/ac902012e. 20166672

[ref35] Tarnapolsky A.; Freger V. Modeling QCM-D Response to Deposition and Attachment of Microparticles and Living Cells. Anal. Chem. 2018, 90, 13960–13968. 10.1021/acs.analchem.8b03411. 30295025

[ref36] Hansen C. M. The Universality of the Solubility Parameter. Ind. Eng. Chem. Prod. Res. Dev. 1969, 8, 2–11. 10.1021/i360029a002.

[ref37] Hansen C. M. The Three Dimensional Solubility Parameter and Solvent Diffusion Coefficient Doctoral Dissertation; University of Copenhagen, 1969.

[ref38] Barton A. F. M. CRC Handbook of Solubility Parameters and Other Cohesion Parameters, 2nd ed.; CRC Press, 2017; pp 422–429.

[ref39] Baglioni M.; Montis C.; Chelazzi D.; Giorgi R.; Berti D.; Baglioni P. Polymer Film Dewetting by Water/Surfactant/Good-Solvent Mixtures: A Mechanistic Insight and Its Implications for the Conservation of Cultural Heritage. Angew. Chem., Int. Ed. 2018, 57, 7355–7359. 10.1002/anie.201710930. 29215783

[ref40] Xu L.; Sharma A.; Joo S. W.; Liu H.; Shi T. Unusual Dewetting of Thin Polymer Films in Liquid Media Containing a Poor Solvent and a Nonsolvent. Langmuir 2014, 30, 14808–14816. 10.1021/la503319w. 25402851

[ref41] Xu L.; Sharma A.; Joo S. W. Dewetting of Stable Thin Polymer Films Induced by a Poor Solvent: Role of Polar Interactions. Macromolecules 2012, 45, 6628–6633. 10.1021/ma301227m.

[ref42] Barton A. F. M. Solubility Parameters. Chem. Rev. 1975, 75, 731–753. 10.1021/cr60298a003.

[ref43] Chernyak Y. Dielectric Constant, Dipole Moment, and Solubility Parameters of Some Cyclic Acid Esters. J. Chem. Eng. Data 2006, 51, 416–418. 10.1021/je050341y.

[ref44] Hanzawa M.; Oohinata H.; Kawano S.; Akamatsu M.; Sakai K.; Sakai H. Removal Mechanism of Photoresist in Alkylene Carbonates with Water and Pluronic Surfactant. J. Jpn. Soc. Colour Mater. 2019, 92, 181–185. 10.4011/shikizai.92.181.

[ref45] Costanzo S.; Di Sarno A.; D’Apuzzo M.; Avallone P. R.; Raccone E.; Bellissimo A.; Auriemma F.; Grizzuti N.; Pasquino R. Rheology and Morphology of Pluronic F68 in Water. Phys. Fluids 2021, 33, 43113 10.1063/5.0049722.

[ref46] Brandani P.; Stroeve P. Kinetics of Adsorption and Desorption of PEO–PPO–PEO Triblock Copolymers on a Self-Assembled Hydrophobic Surface. Macromolecules 2003, 36, 9502–9509. 10.1021/ma034268x.

[ref47] Liu X.; Wu D.; Turgman-Cohen S.; Genzer J.; Theyson T. W.; Rojas O. J. Adsorption of a Nonionic Symmetric Triblock Copolymer on Surfaces with Different Hydrophobicity. Langmuir 2010, 26, 9565–9574. 10.1021/la100156a. 20355719

