# Supplementary material for: Enhanced Removal of Photoresist Films through Swelling and Dewetting Using Pluronic Surfactants
Source: Langmuir. 2023 Oct 5;39(41):14670–9. doi: 10.1021/acs.langmuir.3c02034 (PMC10586462; doi:10.1021/acs.langmuir.3c02034)
Supplement: Supplementary file 1 — la3c02034_si_001.pdf [file la3c02034_si_001.pdf]

Supporting Information

Enhanced Removal of Photoresist Films through Swelling and Dewetting Using Pluronic Surfactants

Masaki Hanzawa[a*], Taku Ogura[a,b], Masaaki Akamatsu[b,c], Kenichi Sakai[b,d*], Hideki Sakai[b,d]

[a] NIKKOL GROUP Nikko Chemicals Co., Ltd., 3-24-3 Hasune, Itabashi, Tokyo 174-0046, Japan

[b] Research Institute for Science and Technology, Tokyo University of Science, 2641 Yamazaki, Noda, Chiba 278-8510, Japan

[c] Department of Chemistry and Biotechnology, Faculty of Engineering, Tottori University, 4-101 Koyama-Minami, Tottori, Tottori 680-8552, Japan

[d] Department of Pure and Applied Chemistry, Faculty of Science and Technology, Tokyo University of Science, 2641 Yamazaki, Noda, Chiba 278-8510, Japan

Corresponding authors: hanmasa@nikkolgroup.com, k-sakai@rs.tus.ac.jp

Content is 9 pages including 10 figures and 3 tables.

Characterization of photoresist film

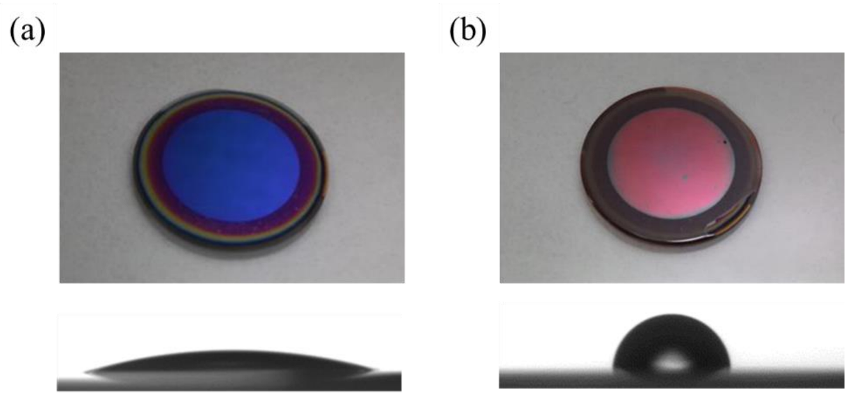

Figure S1. (Upper) Visual representations for (a) a bare QCM-D ITO-sensor and (b) a spin-coated photoresist film on the sensor. (Lower) Water droplets on (a) a bare QCM-D ITO-sensor and (b) a spin-coated photoresist film on the sensor.

Table S1. Photoresist film mass calculated from the Sauerbrey equation (N = 6).

Sensors	Fundamental frequency shift in air (Hz)	Standard errors (Hz)	Coated mass (mg m^{-2})
ITO	-3552.6	12.204	629 $\pm$ 2
Silica	-3318.2	2.0633	587 $\pm$ 0.4

Table S2. Optical properties of the photoresist film on silica substrate for SE measurements.

Wavelength (nm)	Refractive index, *n*	Absorption coefficient, *k*
465	1.6141	0.057168
523	1.6082	0.0089856
599	1.6108	0.0091928
638	1.5962	0.0073586

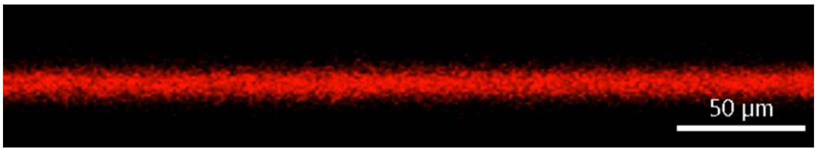

Figure S2. CLSM cross-section representation of the Rhodamine B-labelled photoresist film (red) in air. The scale bar corresponds to 50 μm in length.

Bulk effect in QCM-D measurements

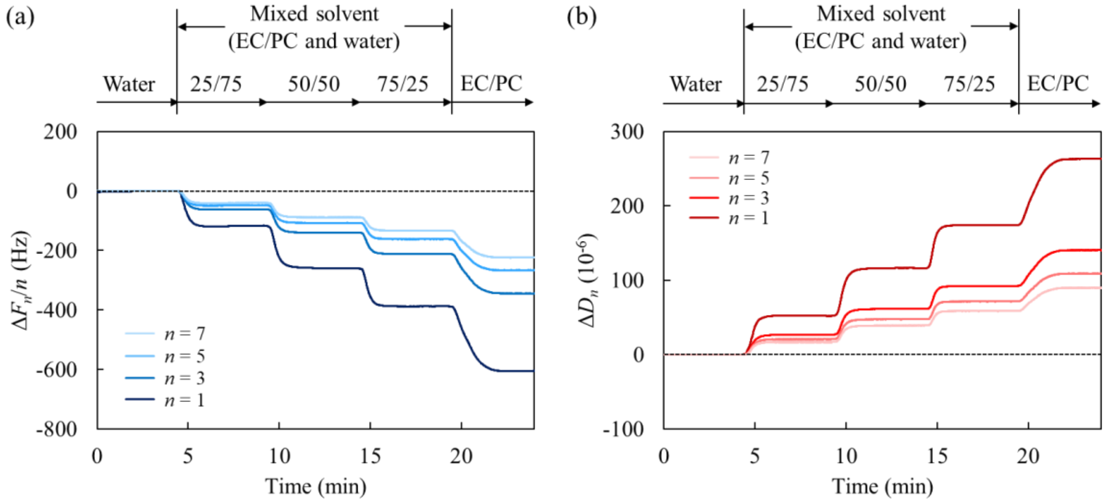

Figure S3. (a) Frequency and (b) dissipation shifts as a function of time of the 1st, 3rd, 5th, and 7th overtones measured for the bulk effect on a pristine QCM-D ITO sensor.

Validation of HSP values for photoresist

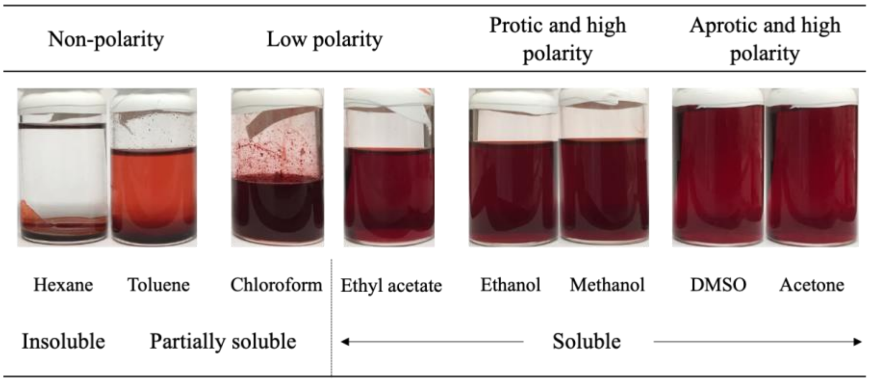

Figure S4. Visual representations of the photoresist in hexane, toluene, chloroform, ethyl acetate, ethanol, methanol, dimethyl sulfoxide (DMSO), and acetone.

Table S3. HSP and calculated RED values of each material for validation.

Materials	δ_{total} [MPa$^{1/2}$]	δ_h [MPa$^{1/2}$]	δ_p [MPa$^{1/2}$]	δ_d [MPa$^{1/2}$]	R_0 [MPa$^{1/2}$]	R_a [MPa$^{1/2}$]	RED
Phenolic resin	27.1	14.6[a]	11.6[a]	19.7[a]	12.7[a]		
Hexane	14.8	0[b]	0[b]	14.8[b]		21.0	1.66
Toluene	18.3	2.05[b]	1.44[b]	18.1[b]		16.5	1.30
Chloroform	18.9	5.74[b]	3.08[b]	17.7[b]		12.9	1.02
Ethyl acetate	18.6	9.23[b]	5.33[b]	15.3[b]		12.1	0.953
Ethanol	26.5	19.4[b]	8.80[b]	15.8[b]		9.58	0.754
Methanol	29.6	22.3[b]	12.3[b]	15.1[b]		12.0	0.945
DMSO	26.7	10.2[b]	16.4[b]	18.4[b]		6.94	0.546
Acetone	19.9	7.00[b]	10.4[b]	15.5[b]		11.4	0.898

[a]From reference 1

[b]From reference 2

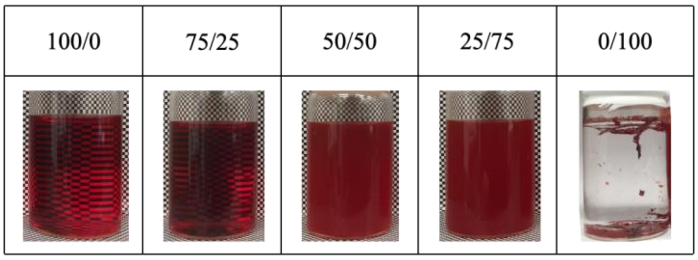

Figure S5. Visual representations of the photoresist in the mixture of EC/PC and water. The solvent weight ratios of EC/PC and water were set at 100/0, 75/25, 50/50, 25/75, and 0/100, respectively.

Removal of photoresist films without Pluronics

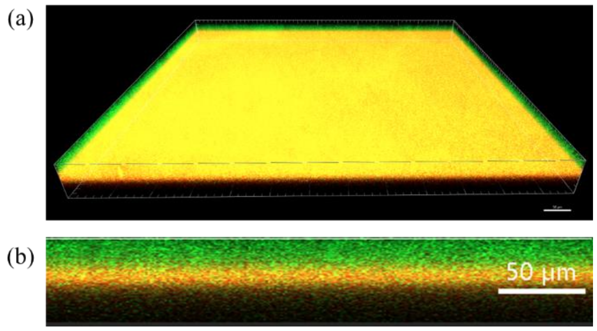

Figure S6. CLSM images of the Rhodamine-B-labelled photoresist film (red) in Rhodamine-110-chloride-labeled EC/PC-to-water (green): (a) 3D overview and 2D cross-section representations. The weight ratio of EC/PC and water was set to 25/75. The scale bar corresponds to 50 μm in length.

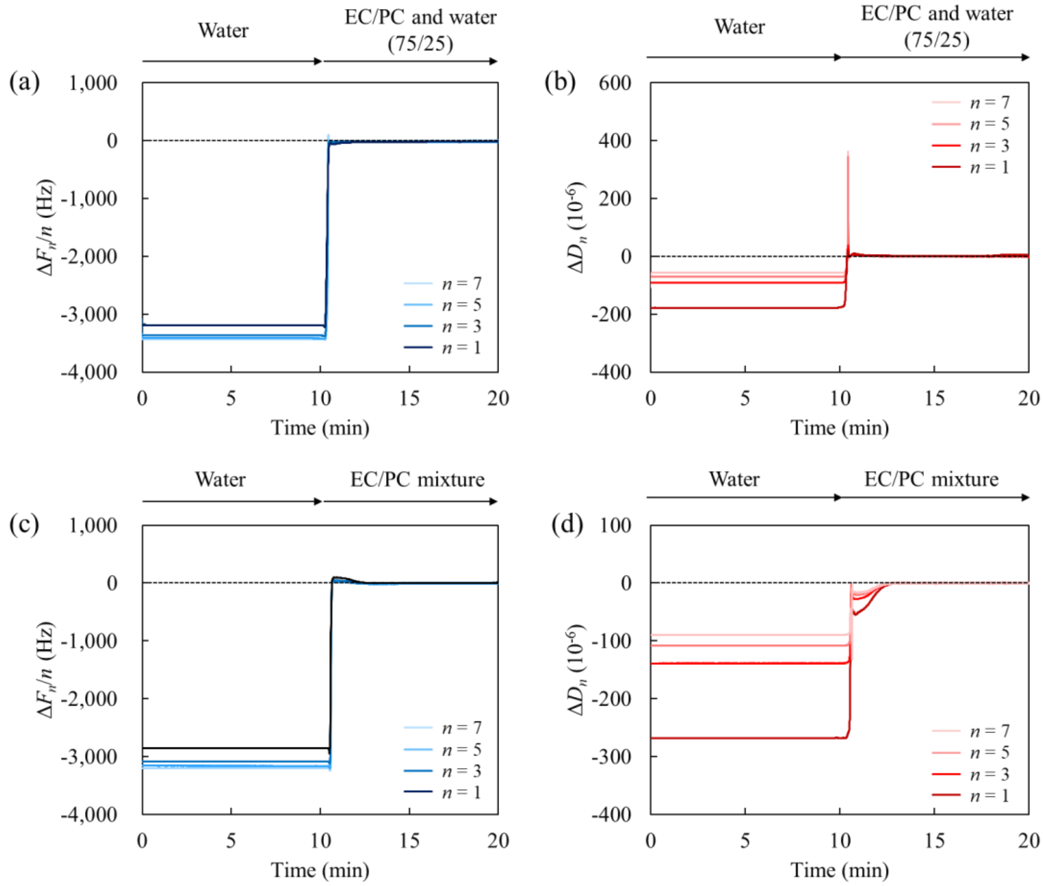

Figure S7. (a,c) Frequency and (b,d) dissipation shifts as a function of time of the 1st, 3rd, 5th, and 7th overtones for the interaction between the photoresist film and (a,b) EC/PC-to-water mixture (75/25 w/w) and (c,d) EC/PC mixture.

Visual representation of photoresist films

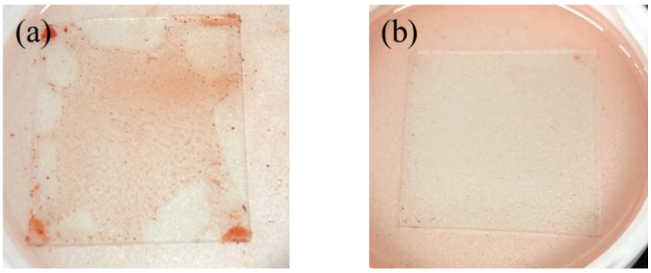

Figure S8. Visual representations of the photoresist film immersed in EC/PC-to-water mixture (a) without and (b) with F-68 (1% w/w). The images represent the photoresist film after immersion for 10 min, and then shaking the immersed substrate. The weight ratio of EC/PC and water was set at 25/75.

Analysis of photoresist particles using ImageJ

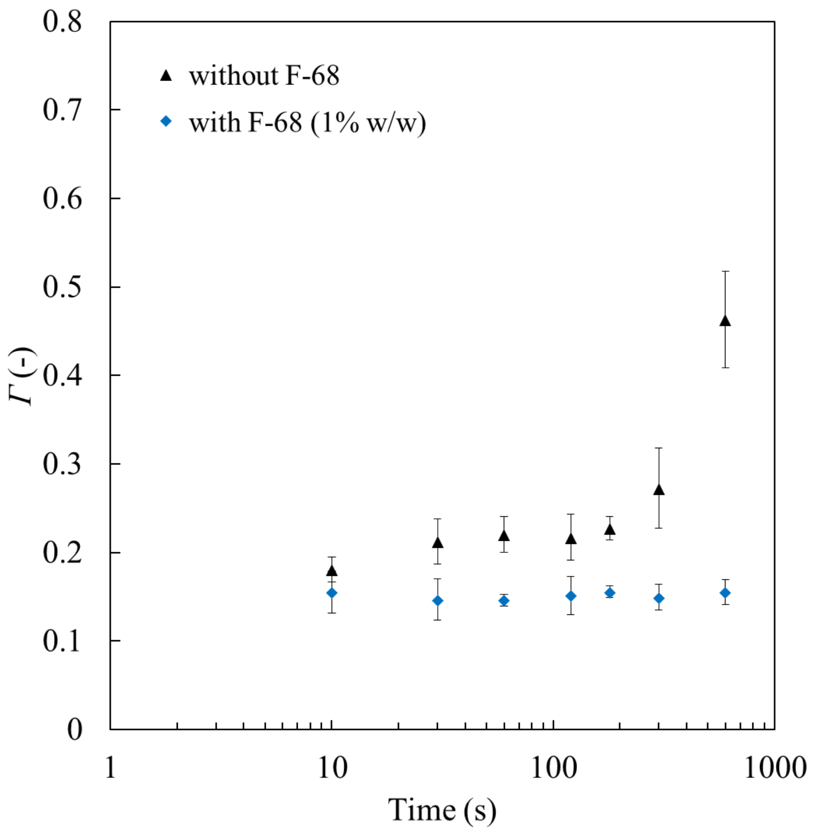

Figure S9. Time-dependent occupied area ratio (*Γ*) of the photoresist particles without and with F-68 obtained in the EC/PC-to-water mixture on the ITO substrate.

Stabilization of photoresist films with F-68

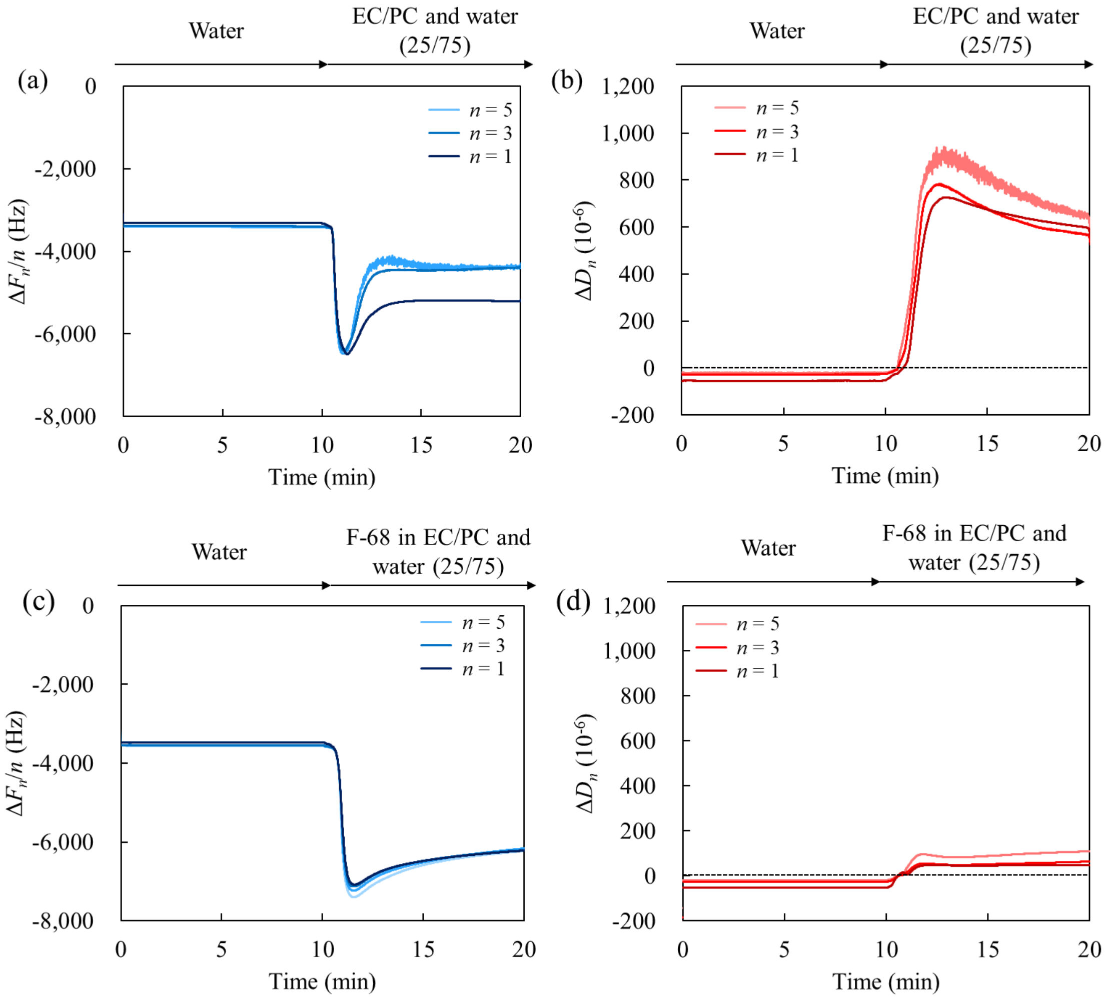

Figure S10. (a,c) Frequency and (b,d) dissipation shifts as a function of time of the 1st, 3rd, and 5th overtones for the interaction between the photoresist film and EC/PC-to-water mixture (a,b) without F-68 and (c,d) with F-68 (1% w/w). The weight ratio of EC/PC and water was set at 25/75.

References

1. Barton, A.F.M. Solubility Parameters, *Chem. Rev.* **1975**, *75*, 731–753.
2. Barton, A.F.M. *CRC Handbook of Solubility Parameters and Other Cohesion Parameters*, CRC Press Inc., **1983**.
